# Potentially repurposable drugs for schizophrenia identified from its interactome

**DOI:** 10.1038/s41598-019-48307-w

**Published:** 2019-09-03

**Authors:** Kalyani B. Karunakaran, Srilakshmi Chaparala, Madhavi K. Ganapathiraju

**Affiliations:** 1 Supercomputer Education and Research Centre, Indian Institute of Science, Indian Institute of Science, Bengaluru, India; 2 Department of Biomedical Informatics, University of Pittsburgh, Pittsburgh, USA; 3 Intelligent Systems Program, University of Pittsburgh, Pittsburgh, USA

**Keywords:** Drug discovery, Computational biology and bioinformatics

## Abstract

We previously presented the protein-protein interaction network of schizophrenia associated genes, and from it, the drug-protein interactome which showed the drugs that target any of the proteins in the interactome. Here, we studied these drugs further to identify whether any of them may potentially be repurposable for schizophrenia. In schizophrenia, gene expression has been described as a measurable aspect of the disease reflecting the action of risk genes. We studied each of the drugs from the interactome using the BaseSpace Correlation Engine, and shortlisted those that had a negative correlation with differential gene expression of schizophrenia. This analysis resulted in 12 drugs whose differential gene expression (drug versus normal) had an anti-correlation with differential expression for schizophrenia (disorder versus normal). Some of these drugs were already being tested for their clinical activity in schizophrenia and other neuropsychiatric disorders. Several proteins in the protein interactome of the targets of several of these drugs were associated with various neuropsychiatric disorders. The network of genes with opposite drug-induced versus schizophrenia-associated expression profiles were significantly enriched in pathways relevant to schizophrenia etiology and GWAS genes associated with traits or diseases that had a pathophysiological overlap with schizophrenia. Drugs that targeted the same genes as the shortlisted drugs, have also demonstrated clinical activity in schizophrenia and other related disorders. This integrated computational analysis will help translate insights from the schizophrenia drug-protein interactome to clinical research - an important step, especially in the field of psychiatric drug development which faces a high failure rate.

## Introduction

Schizophrenia is a complex disorder with a cumulative impact of variable genetic effects coupled with environmental factors^1^. The Schizophrenia Working Group of the Psychiatric Genomics Consortium (PGC) had identified 108 genetic loci that likely confer risk for schizophrenia. Prior to this, around 25 genes were being studied for their association with the disorder^2^. While the role of genetics has been clearly validated by the genome-wide association studies (GWAS), the functional impact of the risk variants is not well understood. Several of the schizophrenia genes, especially those implicated by the GWAS have unknown functions and/or pathways. To discover the functional role of these genes, and promote discovery of novel therapeutics, we had carried out a computational analysis of the protein-protein interactions (PPI) network, or the interactome, of schizophrenia associated genes^3^. The schizophrenia interactome, comprising 101 schizophrenia genes and about 1,900 PPIs, provided valuable results highlighting the functions and pathways tied to schizophrenia genes through their protein interactome^3^. A valuable result from this study was the drug-target interactome that showed a total of 524 drugs targeting 53 proteins in the schizophrenia interactome. Many of these drugs were labeled for therapeutic value for nervous system as expected, but there were several drugs that were labeled for other anatomical systems in the human body.

As drug approvals for psychiatric indications have been facing a high failure rate in the last few years^4^, it would be beneficial to study whether these drugs that target proteins from the schizophrenia interactome could be repurposed for treatment of schizophrenia. Finding alternate uses for approved drugs would be optimal, and such uses are being found in recent years^5–7^.

Diseases are often considered to be driven by an abnormal or perturbed expression of a multitude of genes which together constitute unique differential (gene) expression signatures (DES)^8–12^. Drugs administered to treat these diseases often revert the expression of these genes to their normal levels^13,14^. DES for disease versus normal are quantified using gene expression analysis based on microarrays and RNA sequencing methods, and are deposited in online repositories, which make the data freely available for integrated computational analyses^15^. Similarly, DES for drug-treated versus untreated is made available through Connectivity Map (CMAP)^16^. In order to analyze the suitability of these drugs for repurposing, we build over the results from our previous work on schizophrenia interactome discovery and analysis^3^, utilizing large transcriptomic databases such as CMAP and Gene Expression Omnibus (GEO), and employing a bioinformatics data analysis software suite named BaseSpace Correlation Engine^17^. The approach of repurposing drugs based on the negative correlation of drug-induced versus disease-associated gene expression profiles has resulted in some valuable results in the past. Topiramate, an anti-convulsant drug used in the treatment of epilepsy, was identified to be potentially repurposable for inflammatory bowel disease (IBD), based on the negative correlation of drug-induced profiles extracted from CMAP and disease-associated profile from GEO^18^. They further validated the efficacy of this drug in a rodent model of IBD^18^.

Many genes harboring variants associated with schizophrenia, such as DTNBP1, DAOA, NRG1 and RGS4, show differential gene expression in post-mortem brain samples obtained from schizophrenia patients compared with normal controls^19^. In schizophrenia, it has been pointed out that the effect of genetic variants may, in fact, be reflected on gene expression rather than on the structure of the proteins coded by these genes^20^. Gene expression has been described as a ‘psychiatric endophenotype’ in schizophrenia^19^. A psychiatric endophenotype may broadly be defined as a measurable phenotype, namely, any neuroanatomical, physiological, psychological, biochemical or molecular aspect of brain function, having some definitive disease-associated genetic component, and contributing to a larger behavioral trait such as ‘cognitive dysfunction’ or ‘psychosis’ underlying a complex disorder such as schizophrenia^19^. The ‘definitive genetic component’, in this case, could be a set of disease susceptibility genes harboring sequence variants affecting the expression of the susceptibility genes themselves, or a set of genes differentially expressed in patients compared with healthy subjects. These genes may uncover novel pathways underlying some behavioral trait contributing to disease etiology. For example, it was recently shown that expression of genes associated with immunological processes vary with cognitive performance in familial schizophrenia^21^. So, our method to identify repurposable drugs may be tested on schizophrenia, in which differential gene expression plays a critical role.

## Results

**Figure 1:**
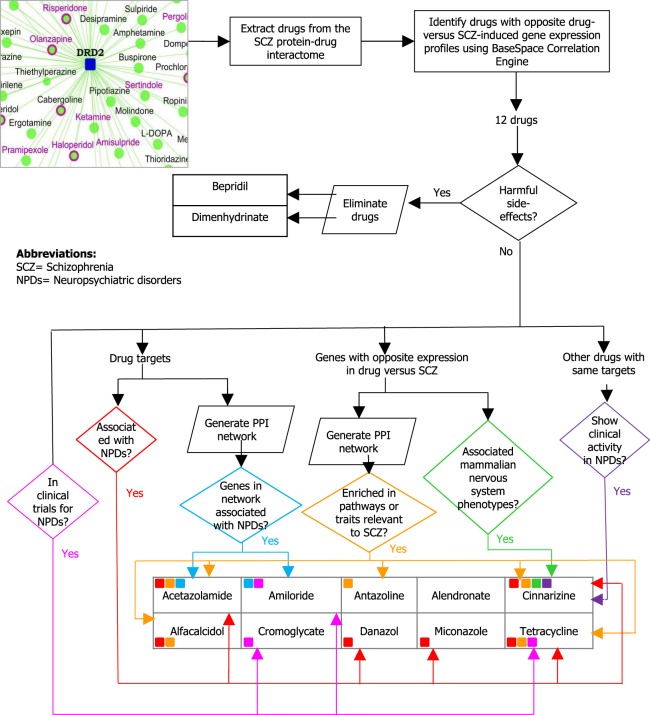
Graphical abstract depicting the steps taken in this study to assess the biological validity of the shortlisted drugs. Drugs were extracted from the schizophrenia drug-protein interactome and screened for negative correlation of drug-induced versus disease-associated gene expression profiles. Drugs shortlisted in this manner were further checked for their toxicity, and eliminated if they were found to have harmful side effects. The targets of the remaining drugs and their network of protein-protein interactions were checked for their association with schizophrenia (SCZ)/other neuropsychiatric disorders (NPDs) using DisGeNET. Genes with opposite expression in drug-induced versus disease-associated profile were analyzed for their association with nervous system phenotypes (Mammalian Phenotype Ontology). Their networks were analyzed for enrichment of SCZ-associated pathways/GWAS traits. Apart from this, it was checked whether the shortlisted drugs are already being tested against NPDs (NIH Clinical Trials), and whether other drugs with the same targets show clinical activity in NPDs. Different sources of supporting information are shown by lines of different colors. Each of the drugs is also tagged with little squares of colors of corresponding supporting information. For example, amiloride is supported by “genes in network associated with neuropsychiatric disorders” (blue) and “in clinical trials for neuropsychiatric disorders” (bright pink). Acetazolamide, cinnarizine and tetracycline each are supported by 3 sources of supporting information.

In our prior work^3^, we presented 524 drugs that target any of the proteins in the Schizophrenia Interactome^3^. We pruned this large list of drugs by comparing differential expression profiles induced by drug to profiles associated with schizophrenia, using our *in silico* protocol, and shortlisted drugs that had a negative correlation between these expression profiles^22^. We carried out bioinformatics analysis on the shortlist of drugs identified thus, to answer the following questions on their biological validity to schizophrenia (see Fig. 1): Have any of these drugs been considered for clinical trials? Are the genes targeted by these drugs associated with neuropsychiatric disorders? Are the genes with opposite expression in drug versus schizophrenia associated with morphological or physiological phenotypes of the mammalian nervous system? Do other drugs targeting the same genes as the shortlisted drugs show clinical activity in neuropsychiatric disorders? Are any genes in the PPI network of the genes targeted by the shortlisted drugs associated with neuropsychiatric disorders? Are any genes in the PPI network of genes with opposite expression in drug versus schizophrenia involved in pathways relevant to schizophrenia? Are they also GWAS genes associated with traits or diseases having a pathophysiological overlap with schizophrenia? These questions were based on the fact that genes associated with traits related to the nervous system and genes linked to neuropsychiatric disorders have been shown to converge in specific co-expression modules, indicating shared genetic basis and disease mechanisms^23^. Drugs used for treatment of a neuropsychiatric disorder may be repurposable for schizophrenia by virtue of shared genes and mechanisms. Each of these sources of information is assessed separately in parallel, highlighting which of the drugs have multiple sources of supporting information.

**Figure 2:**
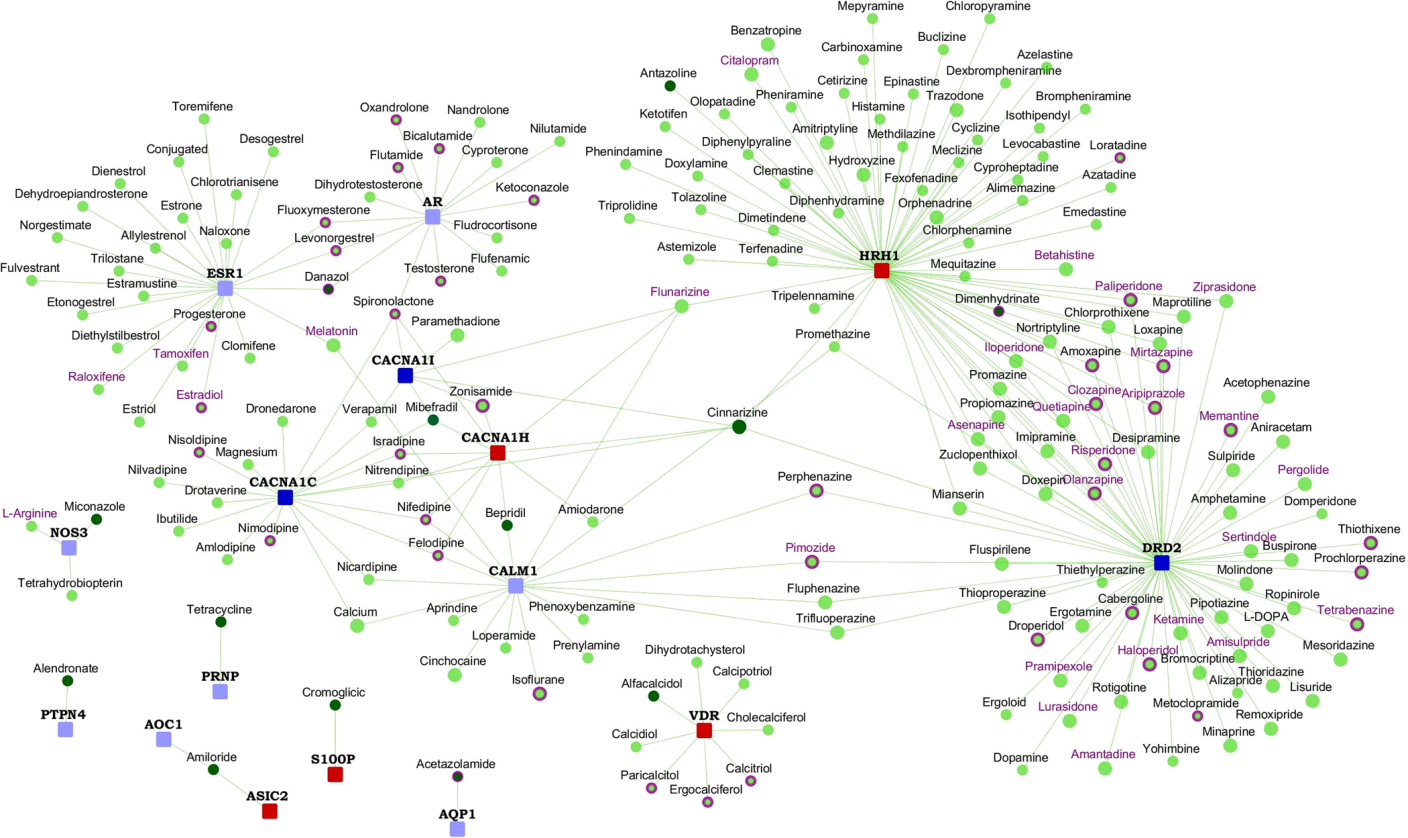
Drugs potentially repurposable for schizophrenia: The network highlights the shortlisted drugs that may be potentially repurposed for schizophrenia. The shortlisted drugs are shown as round nodes colored in dark green, and other drugs are shown as light green nodes. FDA approved drugs are shown with purple borders. Drugs with purple labels are in clinical trials for schizophrenia. Schizophrenia genes are square nodes colored in dark blue, known interactors are colored in light blue and novel interactors in red.

We followed an established approach to identify drugs that have opposite differential expression to the differential expression of schizophrenia (i.e., genes over-expressed in schizophrenia are under-expressed by drug treatment and vice versa)^8^. We identified such drugs using the BaseSpace Correlation Engine software suite, a data analysis platform used to study the effect of diseases and/or drugs on publicly available gene expression data^17^. This analysis resulted in 12 drugs. Although in each case, there are some genes that are differentially expressed in the same direction for both the drug and disorder, the overall effect on the entire transcriptome has an anti-correlation, leading to 12 drugs as potential candidates for further studies (Table 1 and Fig. 2). The top 5 drugs by the score of anti-correlation are cromoglicic acid, bepridil, acetazolamide, dimenhydrinate, cinnarizine, of which bepridil and dimenhydrinate may be excluded due to their side-effects related to nervousness and hallucinations (see Table 1), thus leaving cromoglicic acid, acetazolamide and cinnarizine as top candidates. There were 30 drugs indicated for schizophrenia in DrugBank^24^. 23 out of these occur in the schizophrenia drug-protein interactome (77%). We checked the overlap of drugs indicated for other diseases to infer the specificity of this result, namely, coronary heart disease (25%), lung cancer (50%), diabetes (33%), chronic kidney disease (0%), post-traumatic stress disorder (75%) and bipolar disorder (66%). As expected, there was a larger overlap with neurological disorders compared to other unrelated disorders. 50% overlap with lung cancer drugs may be explained by the large number of drug targets implicated in cancers, and their vital role in numerous basic cellular functions. Eleven of these did not have relevant datasets in BaseSpace, or even though a negative correlation was found, the p-value was insignificant for schizophrenia gene expressions studies. Of 23 *known* schizophrenia drugs – six of them, namely, clozapine, haloperidol, molindone, perphenazine, amitriptyline and nortriptyline, had negative correlation with schizophrenia and 6 others had a positive correlation with schizophrenia. Sources of datasets in which differential expression is observed is listed in Data File 1.

**Table 1:** Details of known schizophrenia drugs and drugs identified as potentially repurposable for schizophrenia: Pharmacokinetic information is collected from DrugBank (www.drugbank.ca). Known schizophrenia drugs are shown in italics.

Drug	Drug class	Original therapeutic purpose(s)	Pharmacokinetic details: dosage form, delivery route, half-life	Toxicity	Correlation with all data types, Overall correlation score	Correlation with SCZ gene expression study, Correlation score	Bs1	Bs2	Bs1 & Bs2 up	Bs1 & Bs2 down	Bs1 up & Bs2 down	Bs1 down & Bs1 up
*Amitriptyline*	Dibenzo-cycloheptenes	Major depressive disorder and anxiety disorders, treatment of secondary depression in schizophrenia	Tablet, oral, 25 hours	Abnormally low blood pressure, confusion, convulsions, dilated pupils and other eye problems on overdosing, and withdrawal symptoms including gastrointestinal disturbances, anxiety, and insomnia	Negative, 76	Negative, 100	HL60 cells + amitriptyline, 12.8 uM _vs_ DMSO vehicle	Hippocampus tissues from schizophrenia patients _vs_ normals	9	3	19	31
*Haloperidol*	Alkyl-phenyl-ketone	Schizophrenia and other psychoses, delusional disorders, ballism, and Tourette syndrome, adjunctive therapy in mental retardation, chorea associated with Huntington’s disease	Solution/tablet, oral, 24 hours	Cardiovascular effects, extrapyramidal symptoms, tardive dyskinesia, neuroleptic malignant syndrome, hematologic effects	Negative, 70	Negative, 68	HL60 cells + haloperidol, 10 μM vs. DMSO vehicle	Prefrontal cortex Brodmann area 46 of schizophreniacs with short DOI vs. helathy controls	3	6	30	1
*Molindone*	Indoles and derivatives	Schizophrenia, other psychoses and aggressive type of undersocialized conduct disorder	Tablet, oral, not available	Not available	Negative, 76	Negative, 57	MCF7 + molindone, 12.8 μM vs. DMSO vehicle	Hippocampus tissues from schizophrenia patients _vs_ normals	22	23	174	21
*Clozapine*	Dibenzo-diazepines	Atypical antipsychotic drug used in schizophrenia	Tablet, oral, 4 to 12 hours	Black-box warning for agranulocytosis	Negative, 59	Negative, 100	HL60 cells + clozapine, 10 μM vs. DMSO vehicle	Prefrontal cortex Brodmann area 46 of schizophreniacs with short DOI vs. helathy controls	10	23	101	2
*Nortriptyline*	Dibenzo-cycloheptenes	Clinical depression, treatment of depressive symptoms in schizophrenia (dose adjustments are necessary to safely use the drug in schizophrenia, as it has been shown to exacerbate psychosis)	Capsule, oral, 26 hours	Cardiac dysrhythmias, severe hypotension, shock, congestive heart failure, pulmonary edema, convulsions, and CNS depression, including coma on overdosing, and withdrawal symptoms include gastrointestinal disturbances, anxiety, and insomnia	Negative, 50	Negative, 89	HL60 cells + nortriptyline, 13.4 uM _vs_ DMSO vehicle	Hippocampus tissues from schizophrenia patients _vs_ normals	6	6	26	3
*Perphenazine*	Phenothiazines	Schizophrenia and the manic phases of bipolar disorder	Tablet, oral, 8 to 12 hours	Stupor or coma, convulsive seizures in children	Negative, 80	Negative, 100	HL60 cells + perphenazine, 10 μM vs. DMSO vehicle	Prefrontal cortex Brodmann area 46 of schizophreniacs with short DOI vs. helathy controls	4	7	78	7
Acetazo-lamide	Thiadiazole sulfonamides	Glaucoma, mountain sickness	Tablet, oral, 3 to 9 hours	Not available	Negative, 76	Negative, 100	MCF7 cells + acetazolamide, 18 uM _vs_ DMSO vehicle	Whole blood from schizophrenic patients _vs_ healthy controls_GPL6947	67	38	95	119
Alendronate	Bisphos-phonates	Osteoporosis	Tablet, oral, 10 years	Damage of oesophagus	Negative, 68	Negative, 57	Heart of rats + ALENDRONIC ACID at 138 mg-kg-d in CMC by oral gavage 5d _vs_ vehicle	Associative striatum tissues from schizophrenia patients _vs_ normals	1	14	12	8
Alfacalcidol	Vitamin D and derivatives	Vitamin D supplement	Capsule, oral, not available	Hypercalcemia	Negative, 46	Negative, 100	Liver of rats + ALFACALCIDOL at 043 mg-kg-d in CMC by oral gavage 1d _vs_ vehicle	Prefrontal cortex Brodmann area 46 - schizophrenics with short DOI _vs_ healthy controls	22	120	125	25
Amiloride	Aminop-yrazines	Hypertension, heart failure, edema	Tablet, oral, 6 to 9 hours	Dehydration and electrolyte imbalance	Negative, 58	Positive, 66	HL60 cells + amiloride, 13.2 uM _vs_ DMSO vehicle	Whole blood from schizophrenic patients _vs_ healthy controls_GPL6947	36	26	14	18
Antazoline	Phenylben-zamines	Nasal congestion, allergic conjunctivitis	Liquid, opthalmic, not available	Not available	Negative, 60	Negative, 91	HL60 cells + antazoline, 13.2 uM _vs_ DMSO vehicle	Whole blood from schizophrenic patients _vs_ healthy controls_GPL6947	5	10	10	24
Bepridil	Phenylben-zamines	Angina	Tablet, oral, 24 to 50 hours	Gastrointestinal problems, dizziness,asthenia, nervousness	Negative, 77	Negative, 40	HL60 cells + bepridil, 10 uM _vs_ DMSO vehicle	Neural progenitors derived from donor stably expressing GFP - schizophrenia _vs_ normal	36	51	73	68
Cinnarizine	Diphenyl-methanes	Motion sickness, vertigo	Tablet, oral, 3 to 4 hours	Drowsiness, skin problems, lethargy, movement problems	Negative, 64	Negative, 100	HL60 cells + cinnarizine, 10.8 uM _vs_ DMSO vehicle	Whole blood from schizophrenic patients _vs_ healthy controls_GPL6947	20	15	21	57
Cromoglicic acid	Chromones	Asthma prophylaxis, aerosol	Solution, oral, 1.3 hours	Cough, nasal congestion, nausea, sneezing and wheezing	Negative, 84	Negative, 64	MCF7 cells + cromoglicic acid, 7.8 uM _vs_ DMSO vehicle	Neural progenitors derived from donor stably expressing GFP - schizophrenia _vs_ normal	15	18	36	13
Danazol	Estrane steroids	Endometriosis, fibrocystic breast disease, hereditary angioedema	Capsule, oral, 24 hours	Not available	Negative, 61	Negative, 100	HL60 cells + danazol, 11.8 uM _vs_ DMSO vehicle	Whole blood from schizophrenic patients _vs_ healthy controls_GPL6947	173	335	460	1264
Dimenhy-drinate	Diphenyl-methanes	Motion sickness, nausea	Solution, intramuscular or intravenous, 1 to 4 hours	Delerium, hallucinations, excitement	Negative, 64	Negative, 50	HL60 cells + dimenhydrinate, 8.6 uM _vs_ DMSO vehicle	Whole blood from schizophrenic patients _vs_ healthy controls_GPL6947	22	10	35	6
Miconazole	Benzylethers	Antifungal medication used in vaginal infections	Tablet, buccal, not available	Oral toxicity in mice at LD50 = 3800 mg/kg	Negative, 60	Negative, 50	HL60 cells + miconazole, 9.6 uM _vs_ DMSO vehicle	Hippocampus tissues from schizophrenia patients _vs_ normals	11	19	66	20
Tetracycline	Tetracyclines	Antibiotic used in acne, cholera, brucellosis, plague, malaria, and syphilis	Capsule, oral, 6 to 12 hours	Oral toxicity in mice at LD50 = 808 mg/kg	Negative, 49	Negative, 100	HL60 cells + tetracycline, 8.4 uM _vs_ DMSO vehicle	Whole blood from schizophrenic patients _vs_ healthy controls_GPL6947	26	20	44	149

### Acetazolamide

**Figure 3:**
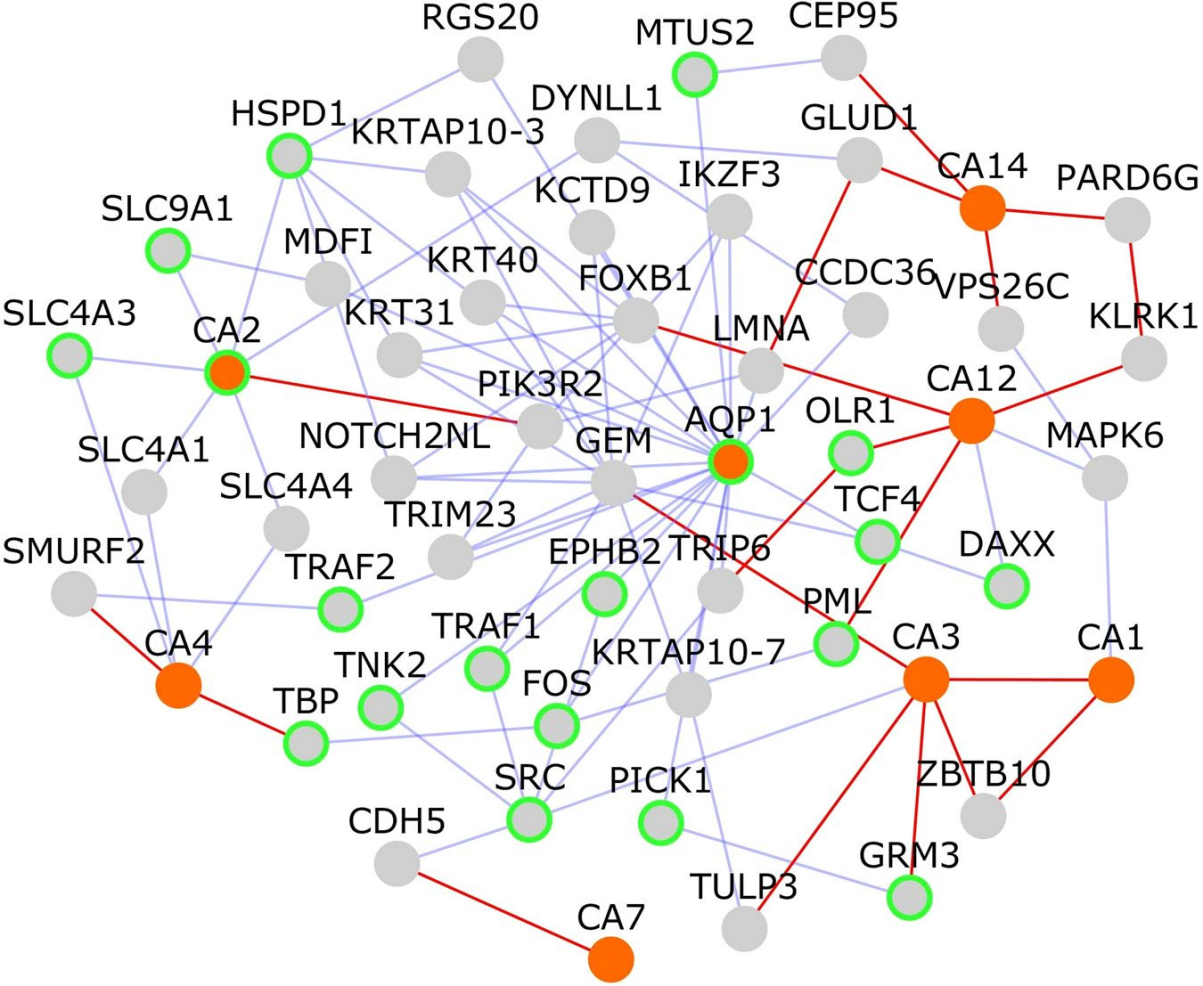
Network of PPIs among targets of acetazolamide: The network shows protein-protein interactions that connect the targets of acetazolamide, which are shown as orange colored nodes. Nodes that connect these target genes are shown as grey colored nodes. Nodes with light green borders are genes associated with neuropsychiatric disorders. Novel interactions are shown as red edges and known interactions as blue edges.

The protein targets of acetazolamide are carbonic anhydrases (CA*) and aquaporin (AQP1). We collected known and computationally predicted PPIs of these targets of acetazolamide and queried the DisGeNet^25^ database whether any of the proteins in this interaction network are associated with neuropsychiatric disorders. While Fig. 2 shows the protein targets only from schizophrenia interactome, Fig. 3 shows the network of all protein targets (orange colored nodes) of acetazolamide and their PPIs. Nineteen genes within this network are associated with various neuropsychiatric disorders (nodes with green border in Fig. 3; Data File 2): AQP1 and CA2, which are acetazolamide targets, DAXX, EPHB2, HSPD1, SLC4A3, SLC9A1, SRC, TCF4, TNK2, TRAF1, TRAF2, MTUS2, PICK1, GRM3, OLR1, TBP, PML and FOS, giving credence to the consideration that it has a potential application to schizophrenia. Acetazolamide has been shown to have high inhibitory activity against human CA2 (hCA II), the ubiquitous cytosolic enzyme (inhibition constant, K_i_ = 12 nM) and human CA7 (K_i_ = 2.5 nM), the brain-specific form of the enzyme^26^. Human CA2 was found to be catalytically highly active (defined in terms of K_cat_/K_m_ for CO_2_ hydration described by two ionizations at pKa 6.2 and 7.5, with a maximum approaching 8 × 10^7^ M^−1^ s^−1^)^27^. K_cat_/K_m_ for human CA2 is 1.5 × 10^8 ^^27^. The increase in extracellular pH which accompanies neural activity is generated by the exchange of external H^+^ for cytosolic Ca^2+^. This process, and its impact on the glutamate receptors, NMDARs, has been shown to be regulated by CA14 in the synaptic microenvironment^28^. On these lines, it is interesting to note that CA3 has been predicted to be a novel interactor of the glutamate receptor, GRM3, mutations in which have been associated with schizophrenia^29^.

We assembled the network of PPIs of genes that are differentially expressed by each of the shortlisted drugs and carried out network and enrichment analysis using a tool called LENS^30^. The networks of genes found to be differentially expressed in acetazolamide, antazoline and cinnarizine, having an anti-correlation in schizophrenia, were shown to be enriched in ubiquitination and proteasome degradation pathways (Data File 3). The ubiquitin proteasome system has been identified as an important pathway in several genetic studies of neuropsychiatric disorders including Alzheimer’s disease, Parkinson’s disease, psychosis and bipolar disorder^31^. Many gene expression studies performed on blood collected from schizophrenia patients, and on post-mortem samples of hippocampus, prefrontal cortex and temporal cortex of patients have pointed at abnormalities in the ubiquitin proteasome pathway, which targets protein for degradation in the cell^31^. Moreover, reduced protein ubiquitination, reduced levels of ubiquitin and ubiquitin-like activases and ligases, were identified in a region of the brain called the left superior temporal gyrus in schizophrenia patients^31^. Left superior temporal gyrus, the volume of which has been shown to decrease in schizophrenia patients, is involved in the development of auditory hallucinations and thought process abnormalities seen in schizophrenia^31^. Interestingly, acetazolamide which has been shown to mediate diuretic effects through its action on AQP1, induces AQP1 ubiquitination, and a proteasome inhibitor reversed its downregulatory action on AQP1^32^. RAD51AP1 and AQR are novel interactors of the calcium channel CACNA1C and the nicotinic receptor CHRNA7 respectively in the schizophrenia interactome, found to have an anti-correlated expression in schizophrenia and acetazolamide treatment. It has been shown that UAF1, an interaction partner of USP1 deubiquitinating enzyme, associates with RAD51AP1, which interacts with RAD51 to mediate homologous recombination repair^33^. NEDD4-1, an ubiquitin ligase, has been shown to promote the sorting of newly synthesized calcium voltage gated channels for proteasomal degradation^34^. Suppression of AQR in HepG2, a liver cancer line, has been shown to inhibit protein ubiquitination^35^. It has been shown that the expression of nicotinic receptors on the cell surface is regulated by the ubiquitin proteasomal system^36^. The networks of genes found to be differentially expressed in alfacalcidol and tetracycline, having an anti-correlation in schizophrenia, were shown to be enriched in the neutrophil degranulation pathway (Data File 3). Degranulating activity of neutrophils has been attributed to dysfunctional permeability of the blood-brain barrier in schizophrenia^37^.

Network of genes which were differentially expressed in acetazolamide and had an anti-correlation with schizophrenia were found to be significantly enriched for association to rheumatoid arthritis (Data File 3). Recently, the reduced prevalence of rheumatoid arthritis observed in schizophrenia patients was attributed to SNPs (single nucleotide polymorphisms) in the HLA region that conferred differential risk for schizophrenia and rheumatoid arthritis^38^. The interactomes of schizophrenia and rheumatoid arthritis genes also showed a significant overlap even outside of HLA genes, and shared common pathways^38^.

### Alfacalcidol

Alfacalcidol targets the protein VDR which was found to be overexpressed in whole blood obtained from schizophrenic patients compared to healthy controls (fold change (FC) = 2.21, p-value = 0.0037)^39^. The network of genes differentially expressed in alfacalcidol was enriched in GWAS genes associated with inflammatory bowel disease (Data File 3). The incidence of schizophrenia has been shown to be high in patients with immune-mediated inflammatory diseases such as inflammatory bowel disease, rheumatoid arthritis and multiple sclerosis^40^.

### Amiloride

With our focus on candidate drugs for repurposing (i.e. those that exhibited a negative correlation to schizophrenia but are not currently labeled for this use), we queried the ClinicalTrials.gov database (https://clinicaltrials.gov/) and found that amiloride is being tested in clinical trials for its efficacy in attention deficit hyperactivity disorder.

**Figure 4:**
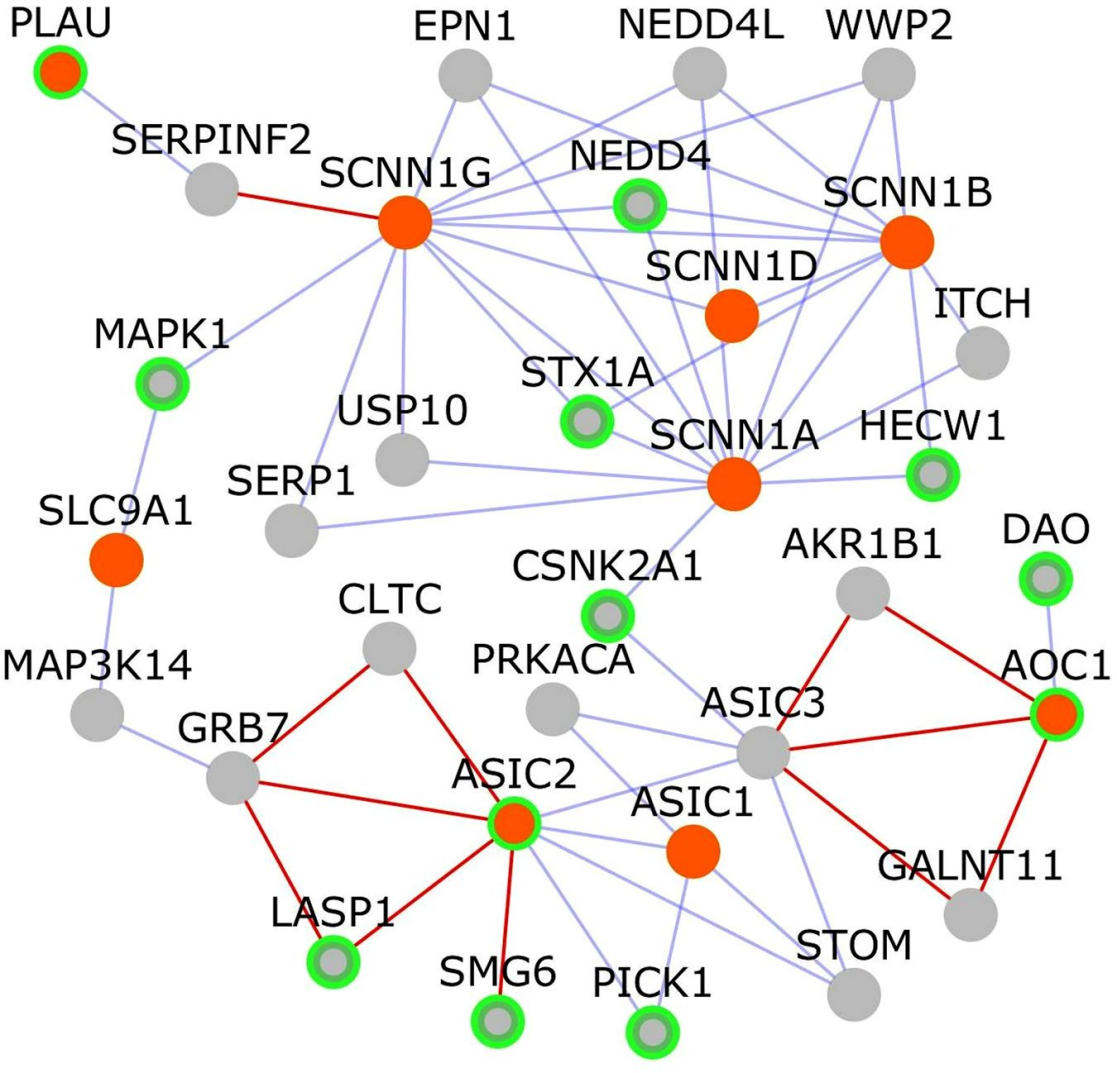
Network of PPIs among targets of amiloride: The network shows protein-protein interactions that connect the targets of amiloride, which are shown as orange colored nodes. Nodes that connect these target genes are shown as grey colored nodes. Nodes with light green borders are genes associated with neuropsychiatric disorders. Novel interactions are shown as red edges and known interactions as blue edges.

We analyzed the PPI network of proteins targeted by the drug amiloride (Fig. 4), despite its positive correlation with schizophrenia gene expression because its overall correlation with a range of schizophrenia datasets was negative, and because of the biological characteristics of its targets. The protein targets of amiloride are ASIC1, ASIC2, AOC1, SLC9A1, PLAU, SCNN1A, SCNN1B, SCNN1G and SCNN1D (orange nodes in Fig. 4). The network of PPIs among these targets of amiloride shows that 12 genes, including ASIC2, AOC1 and PLAU, which are amiloride targets, NEDD4, STX1A, MAPK1, HECW1, DAO, CSNK2A1, LASP1, SMG6 and PICK1 are associated with various neuropsychiatric disorders (nodes with green border in Fig. 4; Data File 2). ASIC2 was a computationally predicted interactor of the gene SMG6, structural variants in which have been associated with schizophrenia or bipolar disorder in a Spanish population^41,42^. SMG6 is located in the chromosomal region 17p13.3, linked to lissencephaly, a neuronal migration disorder arising from incomplete neuronal migration to the cerebral cortex during gestation, and characterized by an absence of normal convolutions in the cerebral cortex and an abnormally small head (or microcephaly)^42,43^. ASICs (*acid-sensing ion channels*) are members of the epithelial Na^+^ channel (ENaC) family of ion channels, expressed in the nervous system^44^. It was shown in a study that ASIC2 is not expressed at the cell surface of high grade glioma (brain tumor) cells and this may be responsible for the constitutively activated inward Na^+^ current, which promotes increased cell growth and migration in these cells^44^. In such glioma cells, compounds such as glycerol and the transcriptional regulator, sodium 4-phenylbutyrate, were shown to inhibit the constitutively activated inward Na^+^ current and reduce cell growth and migration^44^. These compounds were shown to induce the movement of ASIC2 to the plasma membrane, and prevent the active inward current through negative regulatory mechanisms, reducing the ability of glioma cells to proliferate and migrate^44^.

### Antazoline

The networks of genes found to be differentially expressed in antazoline having an anti-correlation in schizophrenia, were shown to be enriched in ubiquitination and proteasome degradation pathways (Data File 3). The network of genes differentially expressed in antazoline, and with an opposite expression in schizophrenia, was significantly enriched in GWAS genes associated with brain connectivity (Data File 3). Abnormal interactions between brain networks have been pointed out to be an important contributing factor in schizophrenia etiology^45^.

### Cinnarizine

The networks of genes found to be differentially expressed in cinnarizine having an anti-correlation in schizophrenia, were shown to be enriched in ubiquitination and proteasome degradation pathways (Data File 3). We checked whether any of the genes having anti-correlated expression on cinnarizine treatment and in schizophrenia were associated with mammalian phenotype ontology (MPO) terms related to various morphological or physiological aspects of the nervous system (http://www.informatics.jax.org/)^46^. It was found that mutations in 13 genes were associated with relevant MPO terms, namely, AHI1, ENTPD1, IFNGR1, NAP1L1, NPTN, PIK3CA, PKN2, PRKDC, PTGS2, RBM12, SEC. 23 A, SS18L1 and UBE3A. Two of these genes, IFNGR1 and AHI1, both linked to ‘abnormal depression-related behavior’, are predicted to have a novel interaction between them. Depressive symptoms have been observed in schizophrenia patients^47^. IFNGR1 has been found to be necessary for the induction of IDO, the tryptophan synthesizing enzyme, which plays a role in depressive behavior, induced by inflammation^47^. AHI1 is associated with susceptibility to schizophrenia and autism^47^. Mice lacking neuronal expression of AHI1 had reduced levels of tyrosine kinase receptor B and a depressive phenotype, which was alleviated by antidepressants and overexpression of TRKB^47^. BDNF/TRKB signaling has been shown to play a key role in depression. Altered BDNF/TRKB signaling in the prefrontal cortex, hippocampus and nucleus accumbens has been shown to give rise to depressive phenotype induced by inflammation^48^. Another gene, UBE3A, was associated with increased dopamine and serotonin levels, abnormal brain wave pattern, cerebral cortex morphology, dendrite morphology, GABA-mediated receptor currents, long term potentiation and nervous system electrophysiology. Yet another gene, NPTN, was linked to abnormal synaptic transmission in the central nervous system and abnormal dendritic spine morphology.

The network of genes differentially expressed in cinnarizine was enriched in GWAS genes associated with inflammatory bowel disease (Data File 3).

We queried Drug Bank^24^ to find drugs that targeted the same genes as the shortlisted drugs, and checked whether they demonstrated any clinical activity in schizophrenia or other neuropsychiatric disorders. Risperidone, nimodipine, nilvadipine, flunarizine, nifedipine, cannabidiol and clozapine target the same genes as cinnarizine. Flunarizine (targeting CALM1, CACNA1H) showed good efficacy and tolerability for the treatment of schizophrenia^49^. Nifedipine (which targets CALM1, CACNA1H) enhanced learning and memory in schizophrenic patients with tardive dyskinesia^50^. Cannabidiol (which targets CACNA1H) shows beneficial effects as an adjunctive drug along with existing anti-psychotic medication in schizophrenia^51^. Risperidone (targeting DRD2) is used to treat schizophrenia, bipolar disorder, and irritability in autistic patients^52–54^. Nimodipine (CACNA1C) has been found effective for treating resistant bipolar mood disorder^55^. Nilvadipine (CACNA1C) was found to be effective in treatment of schizophrenia^56^. Clozapine (targeting HRH1) is effective in treatment-resistant schizophrenia^57^.

Cinnarizine targets CACNA1H which is found to be overexpressed in neural progenitor cells differentiated for 2 days from induced pluripotent stem cells of schizophrenia patients versus healthy subjects (FC = 3.1227, p-value = 4.10E-20)^58^. Cinnarizine targets HRH1, which has been linked to schizophrenia etiology. It also targets CACNA1C, associated with bipolar disorder, schizophrenia and depressive disorder, and CACNA1H, associated with epilepsy and autism. It targets DRD2, linked to bipolar disorder, schizophrenia, depressive disorder, Parkinson’s disease and attention deficit hyperactivity disorder.

### Cromoglicic acid

Cromoglicic acid is being tested in clinical trials for its efficacy in Alzheimer’s disease. It has been reported that cromoglicic acid in combination with ibuprofen reduces the levels of amyloid-beta protein levels, a pathological biomarker in Alzheimer’s disease, and promotes a neuroprotective state by activating microglia and inducing phagocytosis of amyloid-beta proteins^59^. Based on this work, cromoglicic acid has been considered for further study by our clinical collaborators and is currently in clinical trials (ClinicalTrials.gov Identifier: NCT03794076).

### Danazol and miconazole

Danazol and miconazole target ESR1 and NOS3, both associated with Alzheimer’s disease. NOS3 was also identified as a potential target for schizophrenia based on its druggability, membership in schizophrenia-related biological pathways and differential expression in schizophrenia^60^.

### Tetracycline

Minocycline, a broad spectrum tetracycline antibiotic (where tetracycline is one of the shortlisted drugs), has been shown to be effective as an adjunctive drug, improving the effect of antipsychotic drugs in schizophrenia^61^. Tetracycline targets PRNP, linked to depressive disorder, Huntington disease-like 1 and Alzheimer’s disease. The network of genes differentially expressed in tetracycline was enriched in GWAS genes associated with inflammatory bowel disease (Data File 3).

In summary, clinical trial data, network-based analyses and literature review support the biological validity of 9 out of the 12 drugs proposed to be repurposable for schizophrenia, namely, acetazolamide, alfacalcidol, amiloride, antazoline, cinnarizine, cromoglicic acid, danazol, miconazole and tetracycline.

## Discussion

In this section, we discuss cinnarizine and alfacalcidol further due to abundant evidences in literature pointing at their potential utility as repurposable drugs for schizophrenia.

**Figure 5:**
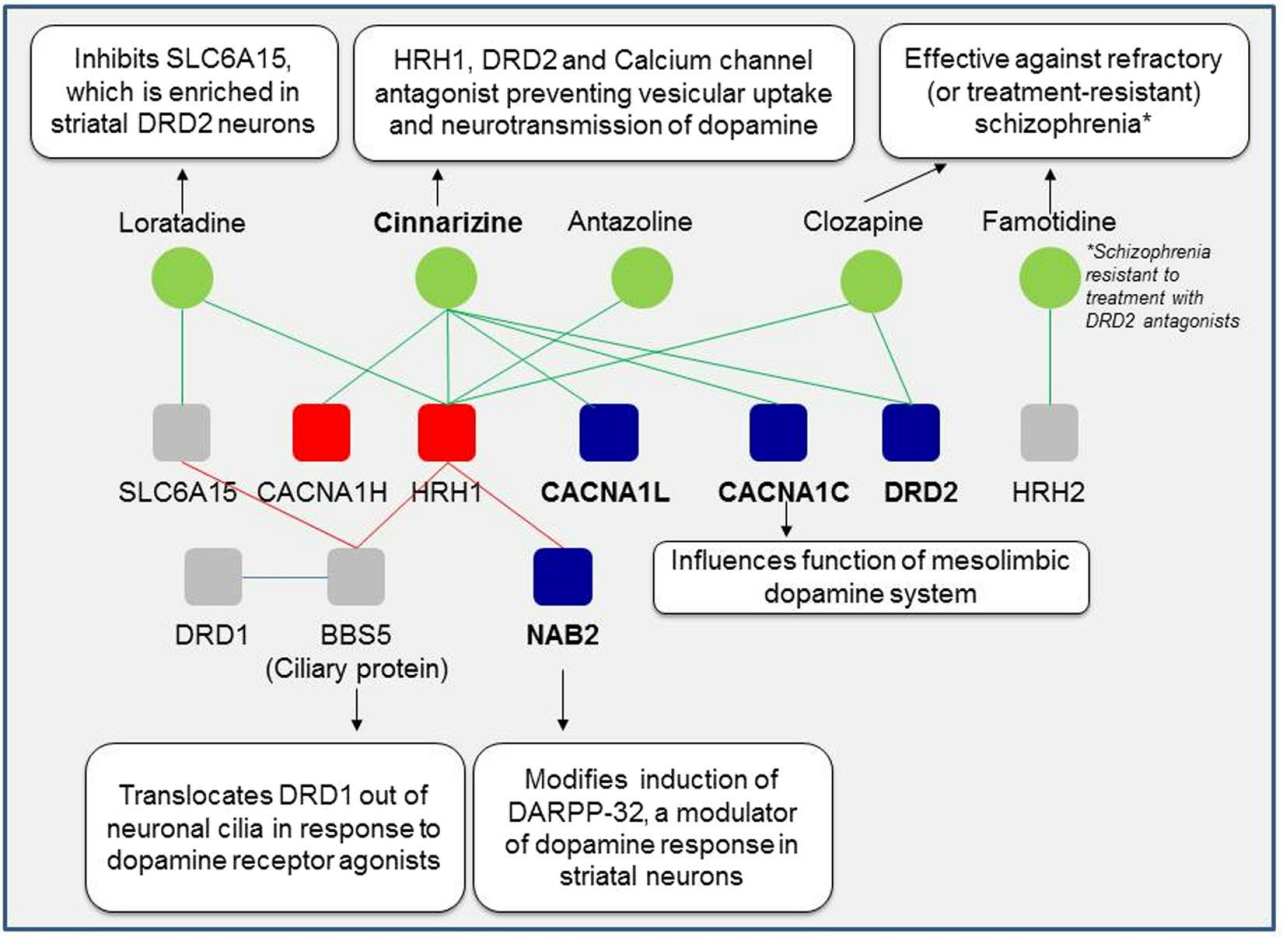
Cinnarizine and its targets in the schizophrenia interactome: The drug cinnarizine is shown here with the proteins it targets from the schizophrenia interactome. 4 additional proteins (BBS5, DRD1, HRH2 and SLC6A15) and 3 additional drugs (loratadine, clozapine and famotidine) that are relevant to the hypothesis are also shown. Cinnarizine, targets 3 schizophrenia genes and 2 novel interactors which constitute calcium channels, and histamine & dopamine receptors. Since histamine antagonists are known to reduce dopamine levels through their action on dopamine receptors, and calcium channel antagonists are known to reduce dopamine neurotransmission, the HRH1, DRD2 and calcium channel antagonist, cinnarizine, may be repurposable for schizophrenia. Another shortlisted drug, antazoline, is not part of the reasoning presented here even though it is an HRH1 antagonist. Schizophrenia genes are shown as dark blue colored nodes, novel interactors are red colored nodes and genes relevant to the hypothesis, which are not in the schizophrenia interactome, are shown as grey colored nodes.

Cinnarizine, an HRH1 (*histamine receptor H1*), DRD2 (*dopamine receptor D2*) and calcium channel antagonist commonly used to treat motion sickness, may be re-purposed to treat symptoms of schizophrenia (see Fig. 5)^62^. Histamine receptors are highly expressed in brain regions associated with the higher cognitive functions disturbed in schizophrenia^63^. Leu49Ser mutation in HRH1 was associated with susceptibility to schizophrenia^64^. Schizophrenia patients have elevated levels of n-tele-methylhistamine, a histamine metabolite, in their cerebrospinal fluid and reduced HRH1 binding in their frontal cortex and cingulate gyrus^65^. According to the revised dopamine hypothesis of schizophrenia, hyperactive dopamine transmission in the mesolimbic areas such as the ventral tegmental area and ventral striatum including nucleus accumbens contribute to disease etiology^66^. Many studies have demonstrated a crosstalk between the dopaminergic and the histamine neuron systems. Compounds acting at histamine receptors have been shown to modulate extracellular striatal dopamine levels^67^. Enhanced release of neuronal histamine was observed on DRD2 activation and in methamphetamine or phencyclidine-induced animal models of schizophrenia^68,69^. Histamine antagonists inhibit behavioral sensitization arising from increased levels of extracellular dopamine^69–72^. The fact that refractory schizophrenia may be treated with clozapine, an HRH1 antagonist, indicates that extra-dopaminergic systems, namely, the histamine neuron system, contribute to schizophrenia etiology^57,69^. Clozapine also exhibits strong affinity to dopaminergic receptors and decreases hyperactivity of the mesolimbic dopaminergic pathway by blocking 5-HT2A (*5-hydroxytryptamine receptor 2A*)^66^. Famotidine, an HRH2 antagonist, significantly reduced psychotic symptoms in schizophrenia patients^73^. The examples of clopazine and famotidine indicate that a drug such as cinnarizine acting as a DRD2 and HRH1 antagonist may serve to alleviate psychotic symptoms arising from the interplay of dopaminergic and histamine neuron systems. Cinnarizine prevents vesicular uptake of dopamine^74^. It shows antagonistic activity at the calcium channel, CACNA1C, whose reduced levels attenuate the function of the mesolimbic dopaminergic pathway and impair behavioral responses to dopamine stimulants^75^. Calcium channel antagonists reduce neurotransmission of dopamine^76^. Even though our computational analysis supports the repurposing of cinnarizine to treat schizophrenia symptoms, its clinical utility can only be validated after experiments in pre-clinical models such as cell lines or animal models, and in clinical trials. Being an anti-histamine, cinnarizine causes drowsiness and its anti-dopaminergic activity may induce Parkinsonism and depression^77^. HRH1, targeted by cinnarizine, was predicted to interact with the schizophrenia gene NAB2. NAB2 modifies the induction of DARPP-32, which modulates the response to dopamine in striatal neurons^78^. HRH1 has also been predicted to interact with BBS5, a ciliary protein. BBS5 interacts with DRD1 and is involved in translocating DRD1 out of the cilia in response to dopamine receptor agonists, thereby implicating neuronal cilia in dopamine signaling^79^. BBS5 was predicted to interact with SLC6A15, which is enriched in striatal DRD2 neurons and inhibited by loratadine, an HRH1 antagonist^80,81^.

The drug alfacalcidol, an analog of vitamin D, commonly used as a vitamin D supplement, or to treat conditions involving imbalance in calcium metabolism such as hypercalcemia and imbalance in bone metabolism such as osteoporosis, may be potentially re-purposed to treat dopaminergic symptoms in schizophrenia, possibly in combination with dopamine receptor antagonists such as clozapine^82,83^. Deficiency of vitamin D exerting its effects through VDR (*vitamin D receptor*) has been observed in schizophrenia patients^84^. Dopaminergic aspects of schizophrenia etiology as proposed by the dopamine hypothesis of schizophrenia may, at least in part, be treated by vitamin D supplementation^66^. In a study based on 9,114 subjects from the Northern Finland 1966 birth cohort, vitamin D supplementation in the first year of life was associated with reduced risk of schizophrenia in males^85^. Several studies have noted an interplay between vitamin D and dopaminergic systems^86^. VDR is highly expressed in brain regions associated with schizophrenia, namely, the hippocampus, prefrontal cortex and dopaminergic neurons in substantia nigra of rats and humans^87^. During early stages of development, VDR is expressed in the mesencephalon precisely at the time when monoamine cells differentiate to dopaminergic cells and dopaminergic systems are innervated^86^. Mice pups with vitamin D deficiency have reduced levels of the enzyme COMT (*catechol-O-methyltransferase*), which converts the dopamine metabolite DOPAC (*3,4-Dihydroxyphenylacetic acid*) into HVA (*homovanillic acid*) and affects the dopamine turnover^86^. In rats with vitamin D deficiency, the effect of MK-801, an NMDA (*N-methyl-D-aspartate*) receptor antagonist which indirectly activates dopaminergic activity and also induces hyperlocomotion in animals, was found to be attenuated with the use of haloperidol, a DRD2 (*Dopamine Receptor D2*) anatgonist^88^. In SH-SY5Y cells routinely used to model neural functions, VDR overexpression resulted in increased dopamine levels, overexpression of TH (*tyrosine hydroxylase*) which is an enzyme involved in the production of the precursor of dopamine called L-DOPA and overexpression of DRD2 whose increased activity has been noted in schizophrenia models, among other regulatory effects on genes associated with the dopaminergic system^89,90^. On treatment of these SH-SY5Y cells with calcitriol, a biologically active form of vitamin D, increased levels of dopamine metabolites such as HVA, increased COMT levels and reduced DRD2 expression were observed^90,91^. Even though there are several studies supporting the efficacy of vitamin D supplementation in treating schizophrenia symptoms^85,92^, several groups have argued that these studies were irreplicable and that randomized controlled trials in larger cohorts would be necessary to ascertain its clinical utility, if any^93^.

In this study, we shortlisted several drugs potentially repurposable for schizophrenia based on the negative correlation of drug-induced versus disease-associated gene expression profiles. Even though this approach has resulted in some valuable results in the past, it has several limitations. The gene expression profiles analyzed in this study were induced by drugs in cancer cell lines^16^, and not in cell lines relevant to schizophrenia. The biological validity of our study will be strengthened if we perform our analysis with gene expression profiles induced by drugs in neuronal cell lines such as SH-SY5Y, in patient-derived induced pluripotent stem cells or in animal models of schizophrenia. However it is to be noted that such data has been shown to be valuable for repurposing drugs even for non-cancer diseases. Specific examples include repurposing of topiramate, an anti-epileptic drug, for inflammatory bowel disease^18^, repurposing of drugs for schizophrenia^94^ and repurposing of drugs for bipolar disorder^95^. These studies show that the drug-induced profiles generated in non-neural cells and deposited in CMAP are amenable to analysis involving neuropsychiatric disorders. Our future analysis will also focus on interrogating gene expression datasets of larger sample sizes. In summary, we showed that the drugs repurposable for schizophrenia may be identified from the schizophrenia drug-protein interactome based on gene expression profiles induced by the drug versus associated with the disease, and augmented our findings with clinical trial data, network-based analyses, and literature review. Through this study, we disseminate this list of drugs potentially repurposable drugs for schizophrenia to the scientific community so as to enable clinical translation of these results.

## Methods

### Identification of potentially repurposable drugs using BaseSpace correlation engine

In an earlier work, we constructed the protein-protein interaction network of schizophrenia genes, and then identified the drugs that target any of the proteins in this interactome^3^. Several of these drugs were known to have therapeutic value for nervous system, but there were several drugs that targeted other anatomical systems in the human body^3^. In this work, as a mechanism of shortlisting drugs for further analysis, we selected those that targeted more than two proteins in the schizophrenia interactome or those that target proteins that are also targeted by many drugs. While the first criterion helps in selecting drugs with the capacity to exert several pharmacological actions, a feature that is critical to targeting a disease as multifactorial as schizophrenia, the second criterion may point in the direction of highly druggable targets. For identifying repurposable drugs, it is essential that we tap into undiscovered regions of the PPI network. So, we also included drugs targeting novel proteins predicted to interact with known schizophrenia-associated genes^96^. Next step involved identifying the drugs that have opposite differential expression to the differential expression of schizophrenia (i.e., genes over-expressed in schizophrenia are under-expressed by drug treatment and vice versa). We studied each of these drugs in comparison to gene expression profiles of schizophrenia by using the software suite called BaseSpace (http://www.nextbio.com/b/nextbio.nb). BaseSpace Correlation Engine is used to study the effect of diseases and/or drugs on publicly available gene expression data^17^. Bioset 1 (‘BS1’) or a particular cell line, tissue or blood sample in which differential expression by drug has been studied was compared with a bioset 2 (‘BS2’), another cell line, tissue or blood sample in which differential expression in schizophrenia patients was studied. A correlation score is generated by the tool based on the strength of the overlap or enrichment, between the two biosets. Additional statistical criteria such as correction for multiple hypothesis testing are applied and the correlated biosets are then ranked by statistical significance. A numerical score of 100 is assigned to the most significant result, and the scores of the other results are normalized with respect to the top-ranked result. We excluded drugs with unacceptable levels of toxicity or undesirable pharmacokinetics.

### Network analysis using LENS

LENS (Lens for Enrichment and Network Studies of human proteins) is a web-based tool which may be used to identify pathways and diseases that are significantly enriched among the genes submitted by users^30^. The LENS algorithm finds the nearest neighbor of each gene in the interactome and includes the intermediate interactions that connect them. LENS then computes the statistical significance of the overlap of genes in the network and genes with annotations pertaining to pathways, diseases, drugs and GWASs, and reports a p-value computed from Fisher’s exact test.

Shortlisted drugs which are being tested in clinical trials against various neuropsychiatric disorders were identified from NIH Clinical Trials (https://clinicaltrials.gov/).

Differential expression of the novel interactor VDR in whole blood obtained from schizophrenia patients was identified from GSE38485^39^, and that of CACNA1H in induced pluripotent stem cells of schizophrenia patients was identified from GSE92874^58^.

Association of the various genes in the network of PPIs among targets of the shortlisted drugs was identified from DisGeNET, a database that integrates human gene-disease associations from expert curated databases and text-mining derived associations^25^.

Drugs that targeted the same genes as the shortlisted drugs were identified from DrugBank (https://www.drugbank.ca/)^24^.

### Preprint publication

An earlier version of this article was deposited into preprint server bioRxiv, where it appeared online on October 13, 2018^97^.

## Supplementary information

Dataset 1

Dataset 2

Dataset 3

## Data Availability

Data sharing is not applicable to this article as no datasets were generated during the current study. Source of data that was analyzed in this study has been described in Methods and Data File 1.
